# Voluminous Omental Inflammatory Myofibroblastic Tumor in an Elderly Man: A Case Report and Literature Review

**DOI:** 10.1155/2015/873758

**Published:** 2015-01-22

**Authors:** Pasquale Cianci, Antonio Ambrosi, Alberto Fersini, Nicola Tartaglia, Vincenzo Lizzi, Francesca Sanguedolce, Antonina Parafioriti, Vincenzo Neri

**Affiliations:** ^1^Department of Medical and Surgical Science, University of Foggia, Luigi Pinto Street 1, 71122 Foggia, Italy; ^2^Department of Pathology, Orthopedic Institute “Gaetano Pini”, Cardinal Ferrari Square 1, 20122 Milan, Italy

## Abstract

Inflammatory myofibroblastic tumor (IMT) is a rare neoplasm of intermediate biologic potential, with uncertain etiology. This tumor occurs primarily in the lung, but the tumor may affect any organ system. A 75-year-old male was evaluated for voluminous palpable high abdominal mass with continuous and moderately abdominal pain, associated with abdominal distension for the last two months. Abdominal computed tomography showed a large (32 × 29 × 15 cm) heterogeneously enhanced mass with well-defined margins. At surgery, the mass originated from the greater omentum was completely excised. Histologically the tumor was a mesenchymal neoplasm in smooth muscle differentiation and was characterized by spindle-cell proliferation with lymphocytes, plasma cells, and rare eosinophils. Immunohistochemically, the tumor cells were positive for vimentin and smooth muscle actin and negative for anaplastic lymphoma kinase. Complete surgical resection of IMTs remains the mainstay of treatment associated with a low rate of recurrence. Final diagnosis should be based on histopathological and immunohistochemical findings. Appropriate awareness should be exercised by surgeons to abdominal IMTs in combination with constitutional symptoms, abnormal hematologic findings, and radiological definition, to avoid misdiagnosed.

## 1. Introduction

Inflammatory myofibroblastic tumor (IMT) is a rare neoplasm of intermediate biologic potential as described in the recent WHO classification [1, 2]. The etiology remains unknown; its true origin has been widely debated regarding its neoplastic or postinflammatory nature. Microscopically these tumors have a pathologic differentiation of dominant spindle-cell proliferation with a variable inflammatory component rich in plasma cells which may mimic plasmacytoma [3]. These lesions are diagnosed as masses relating to their anatomic location [4]. It usually involves the lung [5, 6] and the most prevalent extrapulmonary site is abdominal cavity, with the mesentery and omentum represented for 43% [1, 2]. Other extrapulmonary sites include the heart, head and neck, and soft tissues of the trunk and extremities [7–9]. Differently to pulmonary IMT, which occurs in mid-adulthood, extrapulmonary neoplasms affect children and young adults within the first two decades of life and are rare after 30 years. Females are affected slightly more commonly than males [1, 2, 9]. We present a case of IMT originated from greater omentum in a 75-year-old male, and we review the relevant literature.

## 2. Case Presentation

**Figure 1:**
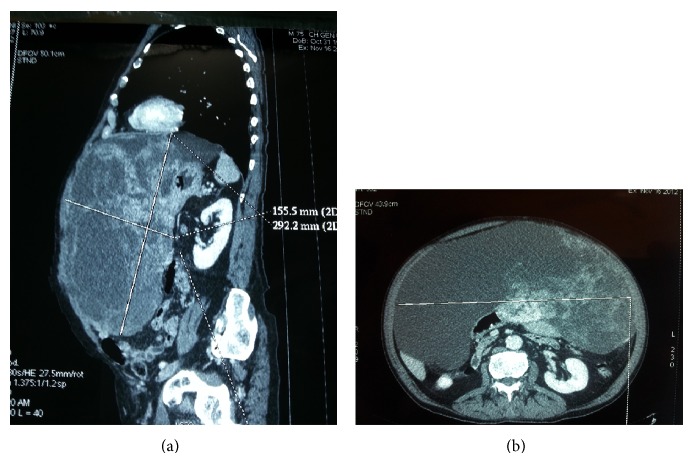
Preoperative contrast-enhanced abdominal CT scan: (a) the sagittal projection shows a large and well-defined, 32 × 29 × 15 cm, heterogeneously enhanced mass that displaces and compresses posteriorly the abdominal organs; (b) the axial projection shows some parts composed of irregular tissue showing enhancement; others are hypodense with cystic aspects and contextual septa.

**Figure 2:**
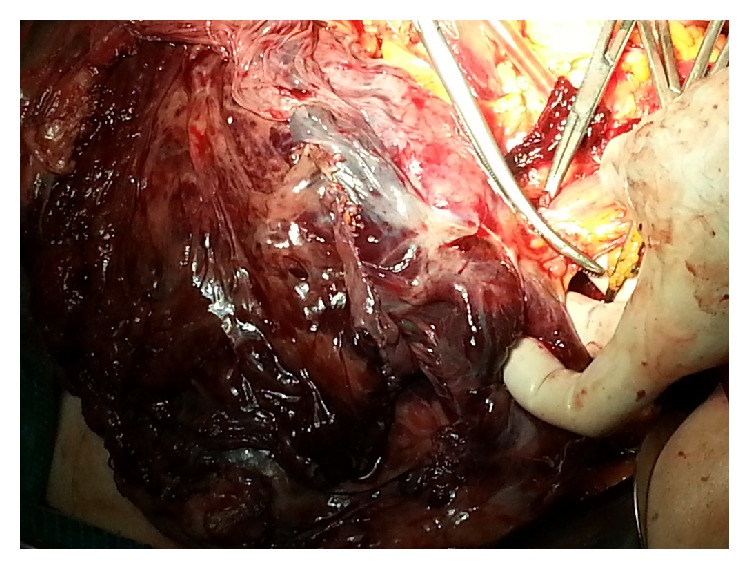
Intraoperative image: laparotomy shows a solid abdominal tumor clearly separated. The mass occupies most of the upper quadrants of the abdomen, originated from the omentum.

**Figure 3:**
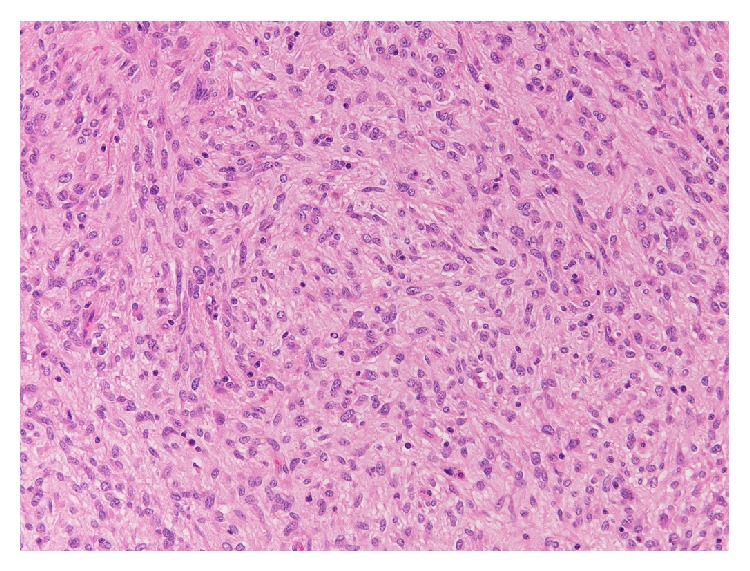
Microscopically image: histological examination shows scattered lymphocytes and plasma cells are admixed with spindle cells, hematoxylin and eosin stain, original magnification 200x.

**Figure 4:**
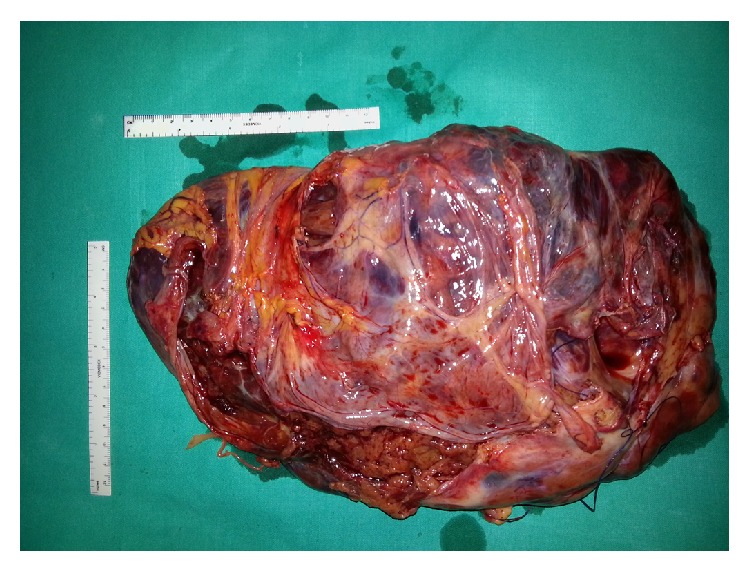
Surgical specimen: voluminous solid lesion measuring 26 cm at the greatest diameter.

A 75-year-old male was admitted to our department presenting with continuous and moderately abdominal pain, associated with abdominal distension for the last two months. On physical examination voluminous palpable mass was revealed in the upper quadrants of the abdomen. Blood analysis showed a mild normocytic, normochromic anemia (RBC: 3.78 × 10^6^/*μ*L, HGB: 11.5 g/dL, and HCT: 34.9%), mild thrombocytosis (PLT: 496 × 10^3^/*μ*L), mild hypergammaglobulinemia (2,4 g/dL), and increased ESR (>20 mm/h). Coagulation tests revealed high values of fibrinogen (460 mg/dL) and fibrinogen degradation products (440 ng/dL). The sonography examination showed an expansive heterogeneous abdominal mass that probably arises from the omental sheets, displacing the diaphragm upwards and bowel loops posterolaterally. Afterwards CT scan was done to characterize the extent of the mass. It showed a large (32 × 29 × 15 cm) heterogeneously enhanced mass with well-defined margins (Figure 1(a)). Some parts of this mass seemed to be composed of irregular tissue showing enhancement; others were hypodense with cyst-like appearance and contextual septa (Figure 1(b)). The lump was displaced posteriorly compressing the abdominal organs. Thus, the radiologist's initial differential diagnosis was gastrointestinal stromal cell tumors (GIST) or sarcoma. Preoperative gastroscopy revealed the presence in prepyloric position of a reepithelialized ulcerative lesion with regular margins (3-4 mm). Biopsy showed moderate lymphoplasmacytic and granulocytic infiltration and flogistic and focal emperipolesis with negativity for the search of* Helicobacter pylori*. Colonoscopy was normal. Body bone scintigraphy for the detection of metastases was negative. A complete surgical excision of the lesion was performed revealing a solid abdominal tumor clearly separated, but in close proximity to adjacent organs (Figure 2). The tumor occupies most of the upper quadrants of the abdomen, displacing the transverse colon downward, in adhesion to anterior wall of the stomach and spleen. The blood perfusion of the tumor was supplied by neoplastic vessels originated from the contiguous organs, mainly from the greater omentum. Macroscopic examination revealed a well-demarcated mass measuring 26 cm at the greatest diameter and grayish color (Figure 3). The capsular surface was smooth. On cut section, the tumor was predominantly solid and sallow, with large cyst-like areas in the periphery with dark brown fluid. Microscopic examination showed a mesenchymal neoplasm benign/borderline (spindle-cell fibromyofibroblastic proliferation with morphological characteristics indicative of low malignant potential), in smooth muscle differentiation, composed of cells with eosinophilic cytoplasm and oval nuclei, with mild and focal nuclear atypia, mixed with elements between lymphocytes and plasma cells and rare eosinophils, in a soft stroma with strong regressive pseudocystic aspects and edematous-hemorrhagic phenomena and vascular dilations (Figure 4). Immunohistochemically, the tumor cells were positive for vimentin and smooth muscle actin (SMA clone 1A4, HHF35) and negative for desmin, S100 protein, calretinin, BLC2, CD117, DOG-1, CD34, CKAE1/AE3, HMB45, MELAN-A, MNF116, and anaplastic lymphoma kinase (analysis FISH with probe ALK Break Apart Vysis, gene ALK 2p23). Based on the immunostains and morphology, a diagnosis of inflammatory myofibroblastic tumor was proferred.

The patient had an uneventful postoperative course and was discharged eight days after the operation. The multidisciplinary team with surgeons and oncologists decided not to proceed with any adjuvant treatment. The patient has been followed up for the last 12 months without clinical or radiological evidence of recurrence.

## 3. Discussion

Inflammatory myofibroblastic tumor is a rare mesenchymal neoplasm of intermediate biologic potential [1, 2], definition recently confirmed by the fourth edition of the WHO classification of tumours of soft tissue and bone [2]. For the first time the IMT was described in the lung in 1939 by Brunn [10]. The lung is the most common location, but these tumors can occur in any organ. Various terms have been used to describe the same lesion including plasma cell granuloma, atypical fibromyxoid tumor, pseudosarcomatous fibromyxoid tumor, postoperative spindle-cell nodules, and inflammatory pseudotumors [1, 2, 9]. Despite the various terms used, IMT is a distinctive lesion composed of myofibroblastic spindle cells accompanied by an inflammatory infiltrate of plasma cells, lymphocytes, and eosinophils [1, 2]. The aetiology is unknown. There are many conflicting opinions regarding the inflammatory or neoplastic nature of these lesions. Some authors believe that it is an immunological response to an infection by organisms (such as Cytomegalovirus Epstein-Barr virus,* Escherichia coli*, interleukin-6 overexpression,* Helicobacter pylori*, herpes simplex virus,* Pseudomonas veronii*, and actinomycetes) or inflammatory process by exaggerated response after abdominal surgery, trauma, radiotherapy, chemotherapy, and steroid use [9, 11–13]. Others suggested that IMTs are true neoplasms based on the role of oncogenic viruses and cytogenetic abnormalities, including ALK gene rearrangements on chromosome 2p23, clonal chromosome abnormalities and DNA aneuploidy [14], and occasional aggressive local behavior along with tumor metastasis [9, 15–18]. The possibility that some of these lesions are neoplastic was discussed almost 50 years ago by Umiker and Iverson [19]; other recent studies on a limited number of cases have demonstrated the presence of clonal cytogenetic abnormalities and ALK expression similar to the anaplastic large cell lymphoma [20, 21]. The current direction seems to favor a neoplastic origin for these tumors, with the potential for malignant progression in a small subset of cases, as described in the recent WHO classifications [1, 2]. In contrast to pulmonary IMT, which occurs in mid-adulthood, extrapulmonary IMT affects children and young adults within the first two decades of life. These tumors show a slight female predominance. Extrapulmonary IMT has a recurrence rate of approximately 25% related to location, resectability, and multinodularity [9]. Rare cases (<5%) also metastasize. Abdominal locations are very variable. Mesentery is the most common site of IMT, with omentum, liver, spleen, colon, stomach, and genitourinary tract [22].

Abdominal IMTs are more confusing from both diagnostic and therapeutic aspects, as they are commonly mistaken for malignant tumors such as peritoneal carcinomatosis, sarcoma, lymphoma, and GIST. Clinically, abdominal IMTs are presented with nonspecific systemic symptoms such as fever, weight loss, malaise, vague abdominal pain, and rarely intestinal obstruction [11]. Specific symptoms depend on the size of the tumor and the mass effect on the adjacent structures. Laboratory findings are nonspecific: hypergammaglobulinemia, increased ESR, thrombocytosis, and anemia [23–26]. The clinical and laboratories findings often do not help in the differential diagnosis; consequently the patients need to perform radiological examinations to determine the nature of the palpable abdominal mass. On sonography they are seen as solid iso- to hypoecoic masses with hypervascularization on Doppler. On CT scan these lesions frequently presented as a well-circumscribed mass or have an infiltrative ill-defined border extending to adjacent organs with variable enhancement characteristics, which depends on the predominance between the cellular component and the fibrous tissue. Calcification, hemorrhage, and necrosis may be found in a minority of cases. The dimensions of the masses vary from 1 cm to greater than 20 cm [27]. On MRI, as discussed by Yagmur et al. [28], IMTs exhibit intermediate signal intensity in T1-weighted images and high signal intensity in T2-weighted images. These lesions are characterized by high ^18^F-FDG PET uptake, similar to malignant tumors; their greater SUV_max⁡_ is attributable to the large number of inflammatory cells within IMTs [28, 29]. In most instances, a definitive diagnosis is made based on the histopathological and immunohistochemical findings performed on the resected tumor. Histopathologically IMT is composed of myofibroblastic spindle cells accompanied by an inflammatory infiltrate of plasma cells, lymphocytes, and eosinophils [1, 2]. Immunohistochemistry better defines the lesion with the presence of vimentin, smooth muscle actin, muscle-specific actin, desmin, and focally for CK and KP-1, in majority of the cases. About 60% of IMTs overexpress ALK proteins which form a specific marker if positive [3, 13]. The presence of chromosomal aberrations (30 to 40% of cases) in these tumors suggests that IMT is a neoplastic proliferation of clonal origin [30] and is associated with more aggressive clinical behavior [3]. Currently, surgical excision with clear resection margins is the treatment of choice for abdominal IMTs. Alternative therapies proposals in cases of incomplete margins have been used but without great success. In the first year, close follow-up is advised, because some patients (15–37%) may have a recurrence, but late recurrence has been reported within 9 years from the time of operation [31]. Our case involves a voluminous abdominal mass arising in an elderly man. The patient presented with moderate abdominal pain and palpable mass in the upper quadrants of the abdomen. Laboratory findings showed a mild anemia, mild thrombocytosis, mild hypergammaglobulinemia, increased ESR, and high values of fibrinogen and fibrinogen degradation products. The sonography examination showed an expansive heterogeneous abdominal mass with hypervascularization on Doppler. CT scan showed a large and well-circumscribed mass with strong heterogeneous enhancement and cystic aspects. A complete surgical excision was performed. The surgical specimen was analyzed and showed an inflammatory myofibroblastic tumor with low potential for malignancy. The tumor was characterized by spindle-cell proliferation with lymphocytes, plasma cells, and rare eosinophils. These cells demonstrated vimentin and smooth muscle actin positivity, absence of ALK reactivity, and low mitotic index. A 1-year follow-up did not indicate metastatic disease or recurrence. In conclusion, IMTs are uncommon neoplasms of intermediate biologic potential that range in most cases from benignancy to the rare aggressive variants. Complete surgical resection of abdominal IMTs remains the mainstay of treatment associated with a low rate of recurrence. Final diagnosis should be based on histopathological and immunohistochemical findings [28]. A careful 1-year follow-up is recommended for early recurrence. Appropriate awareness should be exercised by surgeons to abdominal IMTs in combination with constitutional symptoms, abnormal hematologic findings, and radiological definition, to avoid misdiagnosed.

## References

[1] Coffin C. M., Fletcher J. A., Fletcher C. D. M., Unni K. K., Mertens F. (2002). Inflammatory myofibroblastic tumour. *Pathology and Genetics of Tumours of Soft Tissue and Bone*.

[2] Coffin C. M., Fletcher J. A., Fletcher C. D. M., Bridge J. A., Hogendoorn P. C. W., Mertens F. (2013). Inflammatory myofibroblastic tumour. *WHO Classification of Tumours of Soft Tissue and Bone*.

[3] Coffin C. M., Hornick J. L., Fletcher C. D. M. (2007). Inflammatory myofibroblastic tumor: comparison of clinicopathologic, histologic, and immunohistochemical features including ALK expression in atypical and aggressive cases. *The American Journal of Surgical Pathology*.

[4] Fisher C. (2004). Myofibroblastic malignancies. *Advances in Anatomic Pathology*.

[5] Bahadori M., Liebow A. A. (1973). Plasma cell granulomas of the lung. *Cancer*.

[6] Pettinato G., Manivel J. C., de Rosa N., Dehner L. P. (1990). Inflammatory myofibroblastic tumor (plasma cell granuloma). Clinicopathologic study of 20 cases with immunohistochemical and ultrastructural observations. *American Journal of Clinical Pathology*.

[7] Coffin C. M., Humphrey P. A., Dehner L. P. (1998). Extrapulmonary inflammatory myofibroblastic tumor: a clinical and pathological survey. *Seminars in Diagnostic Pathology*.

[8] Coffin C. M., Dehner L. P., Meis-Kindblom J. M. (1998). Inflammatory myofibroblastic tumor, inflammatory fibrosarcoma, and related lesions: An historical review with differential diagnostic considerations. *Seminars in Diagnostic Pathology*.

[9] Coffin C. M., Watterson J., Priest J. R., Dehner L. P. (1995). Extrapulmonary inflammatory myofibroblastic tumor (inflammatory pseudotumor): a clinicopathologic and immunohistochemical study of 84 cases. *The American Journal of Surgical Pathology*.

[10] Brunn H. (1939). Two interesting benign lung tumors of contradictory histopathology: remarks on the necessity for maintaining chest tumor registry. *The Journal of Thoracic Surgery*.

[11] Estêvão-Costa J., Correia-Pinto J., Rodrigues F. C. (1998). Gastric inflammatory myofibroblastic proliferation in children. *Pediatric Surgery International*.

[12] Shah S. M., Sussman D., Jorda M., Ribeiro A. (2008). EUS with EMR of an inflammatory myofibroblastic tumor of the stomach. *Gastrointestinal Endoscopy*.

[13] Cook J. R., Dehner L. P., Collins M. H. (2001). Anaplastic lymphoma kinase (ALK) expression in the inflammatory myofibroblastic tumor: a comparative immunohistochemical study. *The American Journal of Surgical Pathology*.

[14] Milne A. N. A., Sweeney K. J., O'Riordain D. S. (2006). Inflammatory myofibroblastic tumor with ALK/TPM3 fusion presenting as ileocolic intussusception: an unusual presentation of an unusual neoplasm. *Human Pathology*.

[15] Pungpapong S., Geiger X. J., Raimondo M. (2004). Inflammatory myofibroblastic tumor presenting as a pancreatic mass: a case report and review of the literature. *Journal of the Pancreas*.

[16] Kapusta L. R., Weiss M. A., Ramsay J., Lopez-Beltran A., Srigley J. R. (2003). Inflammatory myofibroblastic tumors of the kidney: a clinicopathologic and immunohistochemical study of 12 cases. *The American Journal of Surgical Pathology*.

[17] Biselli R., Boldrini R., Ferlini C., Boglino C., Inserra A., Bosman C. (1999). Myofibroblastic tumours: neoplasias with divergent behaviour. Ultrastructural and flow cytometric analysis. *Pathology Research and Practice*.

[18] Hussong J. W., Brown M., Perkins S. L., Dehner L. P., Coffin C. M. (1999). Comparison of DNA ploidy, histologic, and immunohistochemical findings with clinical outcome in inflammatory myofibroblastic tumors. *Modern Pathology*.

[19] Umiker W. O., Iverson L. (1954). Postinflammatory tumors of the lung; report of four cases simulating xanthoma, fibroma, or plasma cell tumor. *The Journal of Thoracic Surgery*.

[20] Su L. D., Atayde-Perez A., Sheldon S., Fletcher J. A., Weiss S. W. (1998). Inflammatory myofibroblastic tumor: cytogenetic evidence supporting clonal origin. *Modern Pathology*.

[21] Griffin C. A., Hawkins A. L., Dvorak C., Henkle C., Ellingham T., Perlman E. J. (1999). Recurrent involvement of 2p23 in inflammatory myofibroblastic tumors. *Cancer Research*.

[22] Levy A. D., Rimola J., Mehrotra A. K., Sobin L. H. (2006). From the archives of the AFIP: benign fibrous tumors and tumorlike lesions of the mesentery: radiologic-pathologic correlation. *Radiographics*.

[23] Donner L. R., Trompler R. A., White R. R. (1996). Progression of inflammatory myofibroblastic tumor (inflammatory pseudotumor) of soft tissue into sarcoma after several recurrences. *Human Pathology*.

[24] Stringer M. D., Ramani P., Yeung C. K., Capps S. N. J., Kiely E. M., Spitz L. (1992). Abdominal inflammatory myofibroblastic tumour in children. *British Journal of Surgery*.

[25] Souid A. K., Ziemba M. C., Dubansky A. S. (1993). Inflammatory myofibroblastic tumor in children. *Cancer*.

[26] Sanders B. M., West K. W., Gingalewski C., Engum S., Davis M., Grosfeld J. L. (2001). Inflammatory pseudotumor of the alimentary tract: clinical and surgical experience. *Journal of Pediatric Surgery*.

[27] Sugiyama K., Nakajima Y. (2008). Inflammatory myofibroblastic tumor in the mediastinum mimicking a malignant tumor. *Diagnostic and Interventional Radiology*.

[28] Yagmur Y., Akbulut S., Gumus S. (2014). Mesenteric inflammatory pseudotumor: a case report and comprehensive literature review. *Journal of Gastrointestinal Cancer*.

[29] Hirose Y., Kaida H., Kurata S., Okabe Y., Kage M., Ishibashi M. (2012). Incidental detection of rare mesenteric inflammatory pseudotumor by 18F-FDG PET. *Hellenic Journal of Nuclear Medicine*.

[30] Saleem M. I., Ben-Hamida M. A., Barrett A. M. (2007). Lower abdominal inflammatory myofibroblastic tumor -an unusual presentation—a case report and brief literature review. *European Journal of Pediatrics*.

[31] Bonnet J. P., Basset T., Dijoux D. (1996). Abdominal inflammatory myofibroblastic tumors in children: report of an appendiceal case and review of the literature. *Journal of Pediatric Surgery*.

